# Positive selection, genetic recombination, and intra-host evolution in novel equine coronavirus genomes and other members of the *Embecovirus* subgenus

**DOI:** 10.1128/spectrum.00867-24

**Published:** 2024-10-07

**Authors:** Jordan D. Zehr, Sergei L. Kosakovsky Pond, Stephen D. Shank, Holly McQueary, Jennifer K. Grenier, Gary R. Whittaker, Michael J. Stanhope, Laura B. Goodman

**Affiliations:** 1 Department of Biology, Institute for Genomics and Evolutionary Medicine, Temple University, Philadelphia, Pennsylvania, USA; 2 James A. Baker Institute for Animal Health, College of Veterinary Medicine, Cornell University, Ithaca, New York, USA; 3 Cornell Institute of Biotechnology, Transcriptional Regulation and Expression Facility, Ithaca, New York, USA; 4 Department of Public and Ecosystem Health, College of Veterinary Medicine, Cornell University, Ithaca, New York, USA; Universidade Federal do Rio de Janeiro, Rio de Janeiro, Brazil

**Keywords:** equine coronavirus, natural selection, coronavirus, tropism shift

## Abstract

There are several examples of coronaviruses in the Betacoronavirus subgenus *Embecovirus* that have jumped from an animal to the human host. Studying how evolutionary factors shape coronaviruses in non-human hosts may provide insight into the coronavirus host-switching potential. Equids, such as horses and donkeys, are susceptible to equine coronaviruses (ECoVs). With increased testing prevalence, several ECoV genome sequences have become available for molecular evolutionary analyses, especially those from the United States of America (USA). To date, no analyses have been performed to characterize evolution within coding regions of the ECoV genome. Here, we obtain and describe four new ECoV genome sequences from infected equines from across the USA presenting clinical symptoms of ECoV, and infer ECoV-specific and *Embecovirus*-wide patterns of molecular evolution. Within two of the four data sets analyzed, we find evidence of intra-host evolution within the nucleocapsid (N) gene, suggestive of quasispecies development. We also identify 12 putative genetic recombination events within the ECoV genome, 11 of which fall in ORF1ab. Finally, we infer and compare sites subject to positive selection on the ancestral branch of each major *Embecovirus* member clade. Specifically, for the two currently identified human coronavirus (HCoV) embecoviruses that have spilled from animals to humans (HCoV-OC43 and HCoV-HKU1), we find that there are 42 and 2 such sites, respectively, perhaps reflective of the more complex ancestral evolutionary history of HCoV-OC43, which involves several different animal hosts.

**IMPORTANCE:**

The Betacoronavirus subgenus *Embecovirus* contains coronaviruses that not only pose a health threat to animals and humans, but also have jumped from animal to human host. Equids, such as horses and donkeys are susceptible to equine coronavirus (ECoV) infections. No studies have systematically examined evolutionary patterns within ECoV genomes. Our study addresses this gap and provides insight into intra-host ECoV evolution from infected horses. Further, we identify and report natural selection pattern differences between two embecoviruses that have jumped from animals to humans [human coronavirus OC43 and HKU1 (HCoV-OC43 and HCoV-HKU1, respectively)], and hypothesize that the differences observed may be due to the different animal host(s) that each virus circulated in prior to its jump into humans. Finally, we contribute four novel, high-quality ECoV genomes to the scientific community.

## INTRODUCTION

Coronaviruses (CoVs) that have already jumped, or may have the potential to jump, from animals to humans should be studied to learn the correlates or predictors for the successful colonization of new hosts. Identifying evolutionary factors that may be associated with virulence is also necessary for disease prevention and mitigation. The Betacoronavirus subgenus *Embecovirus* includes CoVs that infect a diverse range of hosts (humans, canines, equids, murines, bovines, lagomorphs, among others) and a range of tissue tropisms (neurological, gastrointestinal, and respiratory) (1). Several of these viruses have jumped from animal to human with minimal apparent genetic changes (2), where recombination events between CoVs from rabbits, camels, and rodents have produced newly emerged embecoviruses (3). Since all human coronaviruses (HCoVs) are hypothesized to have arisen from spillover events involving animals (2, 4), reservoir surveillance and evolutionary analyses of CoVs isolated from animals are critical in mitigating or preventing future human outbreaks.

Equine coronavirus (ECoV) is an *Embecovirus* that infects husbandry animals, such as equines and donkeys (5). The first report of ECoV was in 1999 in a neonatal foal with diarrhea (6) with the virus isolated and named NC-99. Subsequently, ECoV was shown to infect adult horses (7). The ECoV genome encodes seven structural proteins (NS2, HE, S, E, M, N, and I) and upwards of 16 non-structural proteins (NSPs) within ORF1ab (8), one of which, non-structural protein 3 (NSP3) has been found to contain deletion and insertion events that separate ECoV from other embecoviruses (9). Similar to other CoVs, the ECoV Spike protein subunit 1 (S1) is highly antigenic (7). ECoVs follow a fecal-oral route of transmission and have predominantly gastrointestinal tract tropism (10, 11), although respiratory tropism has also been described (12).

As the popularity of horse riding, racing, boarding, and showing has increased, so has the awareness for ECoV testing, and in turn, the prevalence of ECoV has increased (10). While primary ECoV infection is self-limiting and typically does not kill the host (<7% of cases) (13), high case fatality rate (CFR) of ECoV-positive miniature horses and donkeys has been reported (14), where 27% (4/15) animals had either died or been euthanized. Secondary infections can also have high CFR. Hyperammonemia-associated encephalopathy is one such secondary infection reported as a comorbidity of an ECoV infection (10, 14).

Although no human infections have been reported, ECoVs may also pose a potential health risk for both humans and other animals. Recent reservoir surveillance for CoVs has identified research gaps in spatiotemporal sampling in bat samples (15), as well as identified novel CoVs (16–20). Therefore, continued reservoir surveillance for ECoVs may be helpful to study the evolutionary patterns shaping these CoVs. Several key mechanisms of RNA virus evolution include the emergence of quasispecies, which have been shown to yield highly transmissible and pathogenic variants (21, 22), genetic recombination (3, 23–25), and site-specific natural selection (26–29). Here, we apply computational statistical methods to both novel and publicly available ECoV genome sequences to quantify patterns of evolution in ECoVs, as well as across the different CoV members comprising the *Embecovirus* clade. We report evidence of intra-host ECoV evolution in the N gene, identify novel patterns of genetic recombination within ORF1ab, and highlight unique differences in the adaptive evolutionary histories of the ancestral origins of HCoVs.

## MATERIALS AND METHODS

### Clinical horse samples, viral RNA extraction, sequencing, and annotation

Fecal samples from four different horses were collected as part of routine diagnostic care (Table 1). Fecal samples (400 mg subsample) were homogenized in 800 µL phosphate-buffered saline and extracted using the MagMAX Total Nucleic Acid Isolation Kit (AM1840) with a 90-µL elution volume. Elutions were submitted to the Cornell University Transcriptional Regulation and Expression Facility for RNA sequencing. After DNAse treatment and rRNA depletion with the RiboZero HMR kit (Illumina), RNAseq libraries were generated using the NEBNext Ultra II Directional Library Prep kit (New England Biolabs, Ipswich, MA), quality checked, and around 100M reads were targeted for each sample using 2 × 150 bp paired-end sequencing on the Illumina NovaSeq 6000 platform with the S4 flow cell. Relevant metadata, such as age of horse, sex, collection location, and date, as well as clinical symptoms (if reported) and total reads extracted for each data set can be found in Table 1. Genome sequences were annotated based on homology to what has been previously annotated. We also classified non-*Embecovirus* reads with the CZID Illumina mNGS Pipeline v6.8 (30) (https://chanzuckerberg.zendesk.com/hc/en-us/articles/360034790554-Illumina-Pipeline-Details) to identify taxonomic IDs present in each sample. RNAseq data sets can be found under BioProject PRJNA1005627 (each BioSample accession can be found in Table 1), and accession numbers for each of the four novel genomes are as follows: Horse1—OR468097, Horse2—OR468098, Horse3—OR468099, Horse4—OR468100.

**TABLE 1:** Metadata associated with the four horse samples

Horse label and NCBI BioSample	Age, sex, location, date collected	Clinical signs	Total no. of reads obtained from sample
Horse1 (SAMN36993381)	27 years 11 months, Castrated Male, New York, Apr. 2019	Diagnostic request for ECoV (signs not specified)	97,785,256
Horse2 (SAMN36993382)	4 years, Mare, California, Jan. 2018	Fever of 102.4°F, lethargy, anorexia, horse on adjacent property tested ECoV+	144,425,404
Horse3 (SAMN36993383)	16 years, Female, Florida, Jan. 2018	Fever and 3-day history of colitis	108,990,125
Horse4 (SAMN36993384)	17 years Gelding, Florida, Jan. 2020	Fever and respiratory disease	113,401,126

### ECoV genome assembly and intra-host variant calling

Reference-based assembly was performed using the CZID Consensus Genome Pipeline v3.4.4 (30) (https://chanzuckerberg.zendesk.com/hc/en-us/articles/13622345578388-Viral-Consensus-Genome-Pipeline, https://github.com/chanzuckerberg/czid-workflows). All versions of individual tools can be found in that consensus pipeline. Briefly, the automated pipeline quality filters reads, removes reads that map to the designated host (horse), and then uses ECoV genome accession number LC061274.1 to call variants and report a consensus genome (31). The filtered RNAseq data sets and their corresponding assembled genomes were used as input to call intra-host variants with LoFreq galaxy v2.1.5+galaxy0 (32) following a previously published protocol (33). Variants identified with computed minor allele frequencies (mAFs) between 10% and 90%, and coverage (DP) ≥10 (this is to ensure that the variant is found in at least 10 reads) were considered as evidence for quasispecies. This bounded range is informed by previous intra-host coronavirus evolution analyses (34, 35). The corresponding Galaxy workflow (usegalaxy.eu) (36) can be found here: https://usegalaxy.eu/published/workflow?id=862af9fff607117c.

### ECoV genome-wide phylogeny and genetic recombination

We combined the four newly assembled ECoV genomes with the ten publicly available full-length ECoV genomes from GenBank (37). We used MAFFT v7.471 (38) (https://mafft.cbrc.jp/alignment/software/) to generate genome-wide nucleotide alignments from the 14 ECoV genomes and used the Recombination Detection Program v5 (RDP5) (39) to identify evidence and putative boundaries of recombination events. RDP5 employs several methods to detect recombination, and an event was considered supported if at least three methods detected the same event at *P* value < 0.05: RDP (39), GENECONV (40), Bootscan (41), and MaxChi (42). Recombinant segments were removed, and a genome-wide phylogenetic tree was inferred with RAxML-NG v0.9.0git (43) (https://github.com/amkozlov/raxml-ng) under the GTR+Γ nucleotide substitution model and 500 bootstrap replicates. We used SimPlot (44) [SimPlot ++ V1.3 (https://github.com/Stephane-S/Simplot_PlusPlus/releases/tag/v1.3) (45)] to compare and quantify sequence similarity between the available NS2 proteins of ECoV.

### ECoV-specific and *Embecovirus* clade-defining positive selection

We set out to detect signatures of positive selection in two sets of alignments. The first set contained all ECoV protein-coding sequences, and thus was used to infer ECoV-specific signatures of positive selection. The second set contained ECoV sequences, as well as other representative *Embecovirus* Spike protein-coding sequences available in GenBank. The second set was used to infer the history of selection pressure on the ancestral branches leading to each of the respective *Embecovirus* member clades.

In order to generate the ECoV-specific gene alignments, we extracted protein-coding sequences from the 10 publicly available genome sequences on GenBank, the four novel genomes reported herein, as well as used a GenBank homology blast to extract publicly available protein-coding sequences for ECoV hemagglutinin-esterase (HE), spike (S), envelope (E), matrix (M), and nucleocapsid (N). Protein-coding regions from the four novel ECoV genomes were extracted by using reported genome coordinates from previously published and annotated ECoV genomes on GenBank. We generated codon-aware alignments following the procedure available at the Github repository (https://github.com/veg/hyphy-analyses/tree/master/codon-msa) for each ECoV gene. Briefly, in-frame nucleotide sequences were translated and then aligned using MAFFT v7.471. Aligned protein sequences were then mapped back to the nucleotide sequence and a single copy of each unique sequence was retained resulting in the following number of sequences for each coding alignment: HE: 14; S: 16; E: 11; M: 17; and N: 21. Accession numbers used for the ECoV-specific selection analyses performed herein can be found in Table S1.

In order to generate the second set of alignments of ECoV and other representative embecoviruses, we queried GenBank for the most well-represented coding sequence across the virus genera, the Spike sequence. We retrieved Spike sequences for: Betacoronavirus HKU24, BCoV, canine respiratory coronavirus (CRCoV), rabbit coronavirus (HKU14), human coronavirus OC43 (HCoV-OC43), human coronavirus HKU1 (HCoV-HKU1), camel coronavirus (HKU23), porcine hemagglutinating encephalomyelitis virus (PHEV), ECoV, murine hepatitis virus (MHV), and longquan R1 rat coronavirus. All *Embecovirus* accession numbers used are reported in Table S2. *Embecovirus* members with greater than 25 representative sequences [this was the case for HCoVs OC43 and HKU1, as well as bovine coronavirus (BCoV)] were down-sampled following a previously published technique (46). Briefly, a set of sequences was subsampled from each aforementioned *Embecovirus* members with a complete linkage distance clustering algorithm implemented in the TN93 package (https://github.com/veg/tn93). The remaining sequences were then aligned following the codon-aware procedure mentioned above. The unique number of sequences retained for each member can be found in Table S3. Given the high sequence divergence across embecoviruses we used a heuristic procedure to identify and mask potentially misaligned sites in the Spike alignment. We inferred a maximum-likelihood phylogenetic tree with RAxML-NG v0.9.0git under the GTR+Γ nucleotide substitution model and used a sliding window approach over sequences in the tree to examine the proportion of residues in the current window that are not identical that may be introduced through multiple-hit nucleotide mutations. As a heuristic to reduce false positive inferences of natural selection, columns were masked at a cutoff of 0.4. Relevant code can be found here: https://github.com/veg/hyphy-analyses/tree/master/find-outliers.

Both the ECoV-specific and the Spike *Embecovirus*-wide codon-aware alignments were screened for the presence of genetic recombination with the Genetic Algorithm for Recombination Detection (GARD) method (47) to search for the optimal number and location of putative recombination breakpoints based on the small sample Akaike information theoretic criterion. If GARD identified supported recombinant breakpoints, then alignments were partitioned accordingly, and a maximum likelihood phylogeny was inferred for each partition as described previously. Where necessary, phylogenetic branches for downstream selection analyses were labeled with *phylotree.js* (48). Codon-aware alignments along with their respective phylogeny were used as input for downstream natural selection analyses.

**Fig 1:**
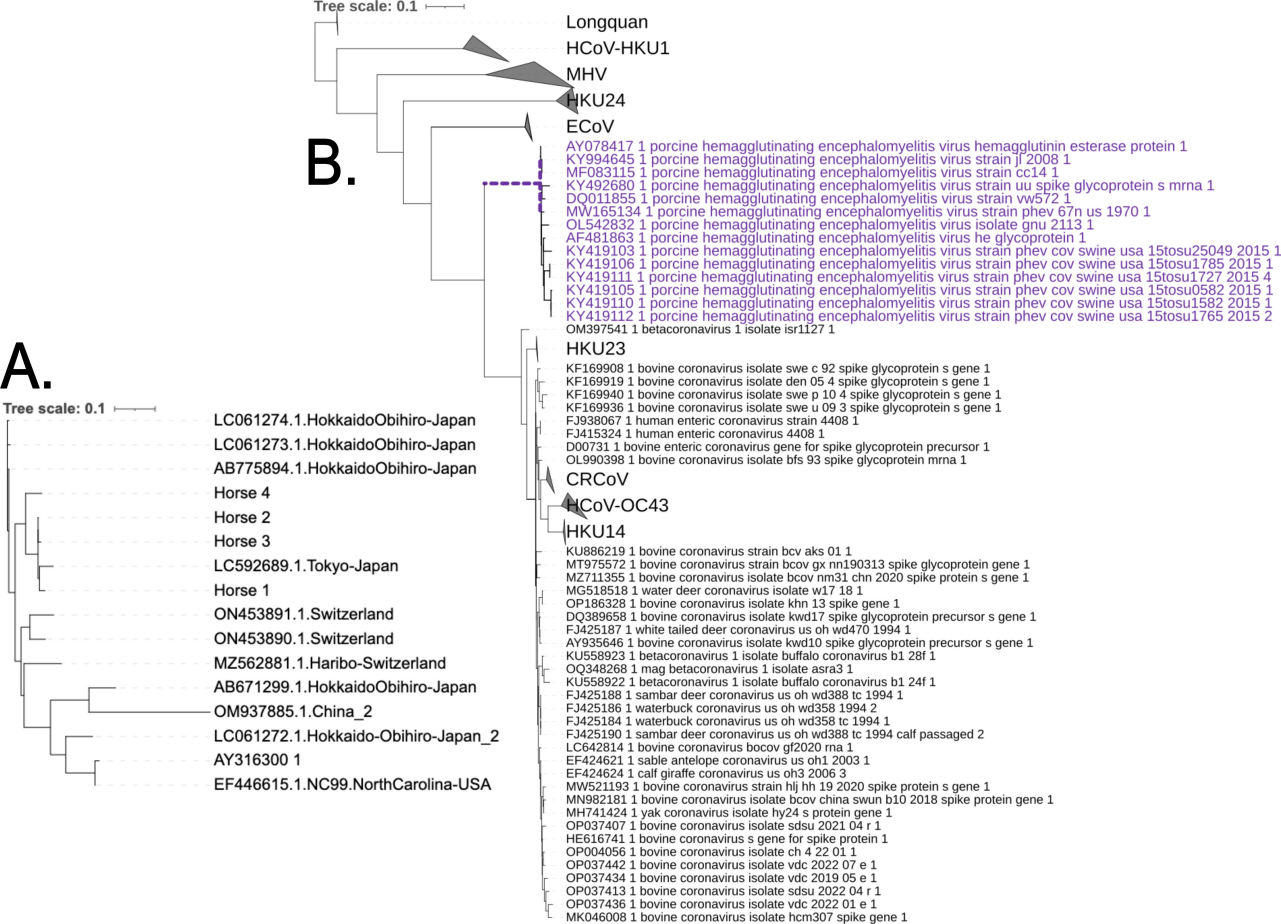
Phylogenetic tree inputs to MEME analyses. Figure (A) is the ECoV-specific spike tree used to infer ECoV-specific selection; all branches were tested. This process was repeated for all ECoV-specific protein-coding sequences analyzed herein. Figure (B) demonstrates how MEME was used to test for the presence of site-specific positive selection on branches of the phylogeny inferred from alignment set two, containing ECoV and other representative *Embecovirus* member sequences. In the example above, the PHEV ancestral branch (dashed purple line) was selected, and MEME tested for the presence of positive selection on that branch. This process was repeated for all 11 *Embecovirus* member clades analyzed herein. Several clades have been collapsed and labeled accordingly. All other trees and associated alignments used for the MEME analyses can be found here: https://data.hyphy.org/web/ECoV/ECoV-data/. Trees are displayed with iTOL (52).

**Fig 2:**
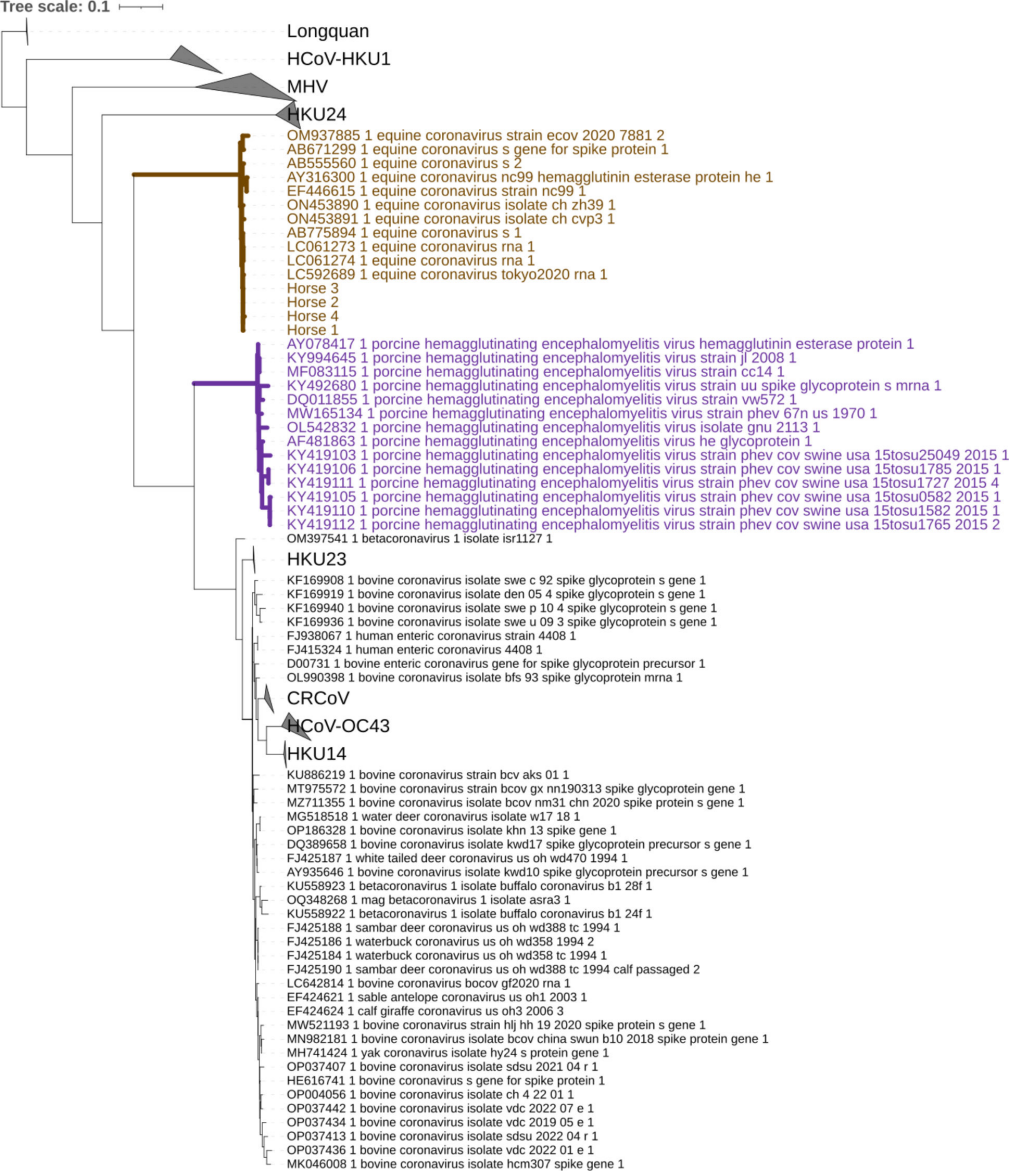
An example phylogenetic input for the Contrast-FEL analyses. ECoV and PHEV sequences in trees inferred from each of the four RFPs from alignment set two were analyzed. These two branch sets were used to test for codon-specific selection pressure differences between ECoV and its sister taxon PHEV. Several clades have been collapsed and labeled accordingly. The three other trees and associated alignments used for the Contrast-FEL analyses can be found here: https://data.hyphy.org/web/ECoV/ECoV-data/. This tree is displayed with iTOL (52).

The Hypothesis testing using Phylogenies (HyPhy) v.2.5.43 software package (49) was used to infer various signals of selection. The Mixed Effects Model of Evolution (MEME) method (50) was used to detect site-specific signals of positive selection. This method is applicable to either all branches in a phylogeny, or a user-defined subset of branches. We used MEME to infer signals of positive selection acting on specific codons in two integral ways. First, the method was used to identify ECoV-specific patterns of positive selection by applying this method to all ECoV gene alignment recombinant free partitions (RFPs)—to all sequences and branches contained within. Second, MEME was applied to the second set of alignments, containing ECoV and other representative *Embecovirus* Spike sequences, to infer site-specific selection on the ancestral branch of each *Embecovirus* clade within the phylogeny. This second application of MEME was used to identify any clade-defining positive selection for each CoV member of the *Embecovirus* subgenus. Examples of how MEME was applied in both applications can be found in Fig. 1. Due to the relatively small number of branches used in the latter MEME analyses (<10), 100 parametric bootstrap replicates were employed; this procedure is computationally expensive, but improves statistical robustness (29). Sites under positive selection with a *P* value ≤0.05 are reported. Phylogenies of the second alignment set, support PHEV as a sister-taxa to ECoV. We used the Contrast-FEL method (51) to test site-specific selection pressure differences between ECoV and its sister taxa (PHEV). An example of how Contrast-FEL was applied to the second set of data can be found in Fig. 2. Codon positions subject to selection in ECoV, along with ECoV-specific nucleotide RFP boundaries correspond to ungapped positions in the ECoV strain NC99 genome (accession number EF446615.1). All alignments and phylogenies used in this study can be found at https://data.hyphy.org/web/ECoV/ECoV-data/.

## RESULTS

### ECoV genome analyses: assemblies, intra-host variants, phylogenetic inference, and genetic recombination

Four full-length ECoV genomes were assembled from RNAseq data sets (containing nearly 100 million reads each) from four horses presenting clinical signs of ECoV, which resulted in an average of 9,078.9× coverage for each assembled genome (see Materials and Methods for details). A genome alignment containing the 4 newly assembled genomes and 10 publicly available full-length ECoV genomes demonstrates that the four newly assembled genomes align from the 5′ to 3′ ends of the genome. We used the assembled genomes and associated RNAseq data sets to test for the presence of intra-host genetic variants (see Materials and Methods for details), indicative of viral quasispecies (21, 53). Two of the four RNAseq data sets had evidence of intra-host genomic variants (Horse 1 and Horse 4; Table 1), where the estimated mAF was between 10% and 90% and coverage depth (DP) greater than 10. Both variants fell within the N gene, where the variant in Horse 1 encoded a non-synonymous C to T transition from serine (S) to phenylalanine (F) and the variant in Horse 4 resulted in a synonymous C to T transition mutation (Table 2).

**TABLE 2:** ECoV intra-host variants^*a*^

Horse genome	Nuc. pos in ref genome	minorAF (mAF)	DP (coverage)	pos in NC99	ref	alt	Protein (AA position)	AA Impact
Horse 1	29,326	10.16	5,510.0	29,406	C	T	N (15)	S → F
Horse 4	29,834	15.16	2,618.0	29,917	T	C	N (185)	Y → Y

^*a*^ All variants are reported with minor allele frequency (mAF), where the mAF refers to the frequency of the “alt” position, and has a depth (DP) ≥10. The “ref” is the nucleotide that appears in the respective genome. The “Protein” column highlights the ECoV protein that the variant appears, with the amino acid position impacted in parentheses and noted by the single letter abbreviation.

**Fig 3:**
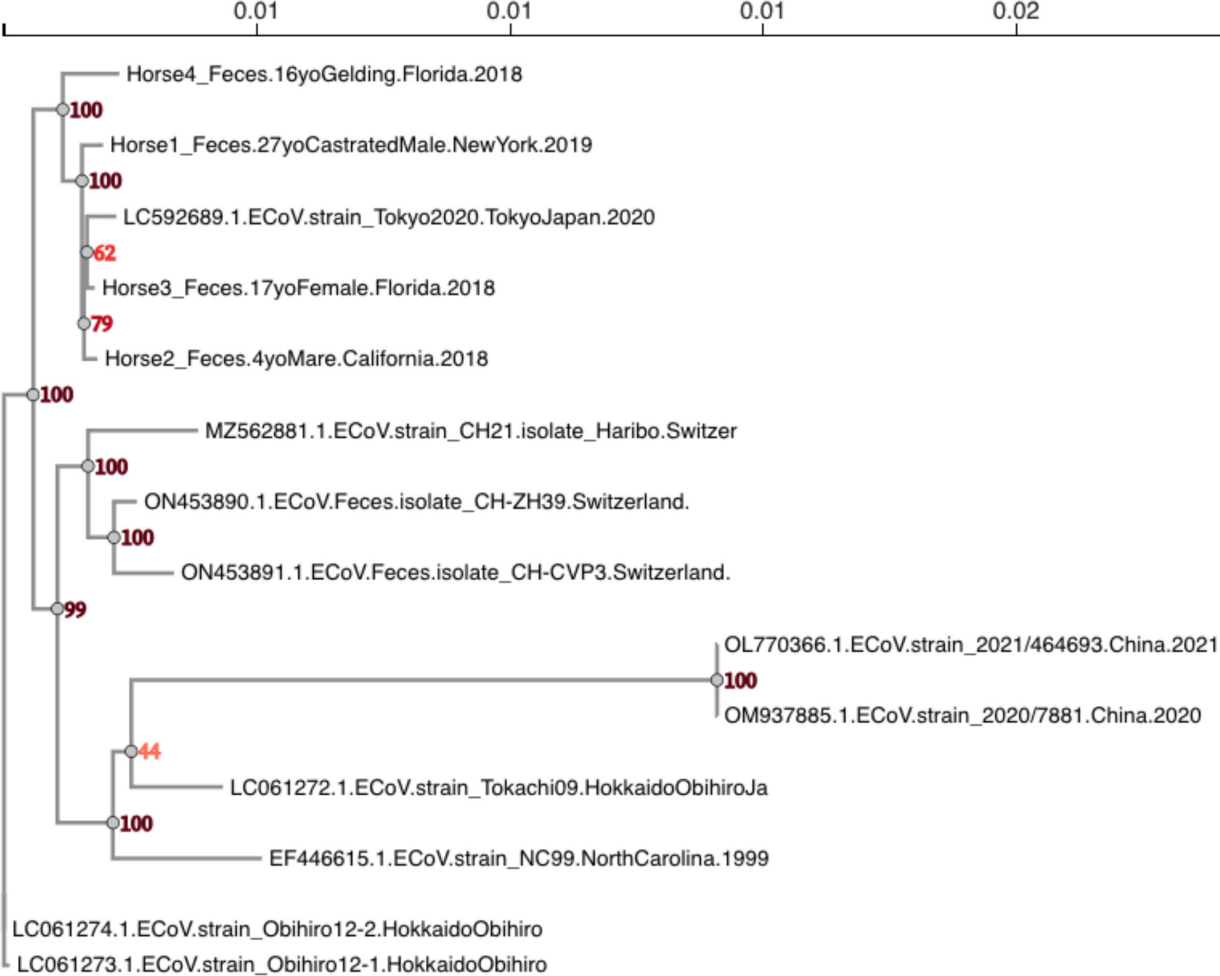
An unrooted, ECoV-specific genome-wide phylogeny reconstructed from full-length genome sequences using RAxML-ng (ML) with 500 bootstrap replicates (bootstrap values are denoted at each node). All recombinant fragments within the genome were identified using RDP5 and then removed, in order to infer a recombinant free phylogeny. This phylogeny demonstrates the intra-taxon relatedness from all publicly available ECoV genomes. Leaf nodes are annotated with accession number and collection location.

A recombination-free, genome-wide maximum likelihood (ML) phylogenetic tree with 500 bootstrap replicates was inferred from 14 ECoV genome sequences (10 publicly available and the 4 novel genomes reported herein) (Fig. 3). The four novel ECoV genomes isolated from horses in North America cluster together, along with a genome sequence isolated from Tokyo (accession number LC592689). Two ECoV genome sequences have been isolated from China and may represent potentially “novel ECoV variants” partially due to a recombinant region around the NS2 gene (5). Indeed, a divergence matrix, estimated by MEGA11 (54, 55), of the NS2 gene from these two Chinese sequences compared to all other publicly available ECoV NS2 sequences, demonstrates they are very clearly the most divergent sequences in the group (Table S4). A similarity plot generated with SimPlot++ (45) also supports the conclusion that the NS2 sequence from both Chinese sequences is dissimilar to the others analyzed (Fig. S1) likely due to the recombination event.

**Fig 4:**
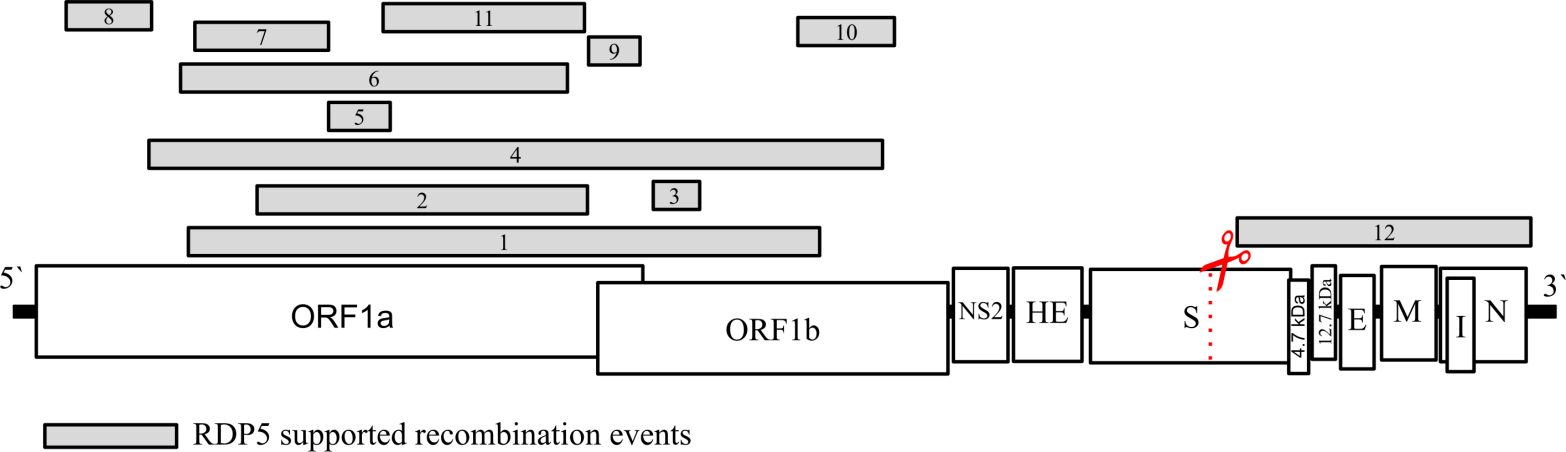
All 12 supported ECoV-specific genome-wide recombination events (numbered gray rectangles) as identified by RDP5 mapped. Events 1 through 11 fell within the ORF1ab boundary, and Event 12 began in the S2 subunit of Spike and continued until the end of the genome. ECoV genes represented include (nucleotide boundaries in parentheses as identified in accession no. EF441165.1): ORF1a (210..13478), ORF1b (13479..21595), NS2 accessory protein [NS2] (21610..22446), Hemagglutinin-esterase protein [HE] (22458..23729), Spike protein [S] with the putative cleavage motif highlighted in a red dashed line with red scissors (23744..27835), 4.7 kDa protein (27825..27947), 12.7 kDa protein (28076..28405), Small envelope protein [E] (28392..28646), Membrane protein [M] (28661..29353), Nucleocapsid [N] (29363..30703), and I protein [I] (29424..30044).

Abundant evidence exists to support recombination histories in CoVs (3, 5, 23–25, 56, 57). The RDP5 package (39) identified 12 supported genome-wide recombination events (see Materials and Methods for details, and Table S5). Eleven of the 12 supported recombination events detected fell within ORF1ab. Event 12 is the only supported recombination event identified outside the ORF1ab region, which begins in subunit 2 (S2) of Spike and continues until the end of the genome (Fig. 4). The RDP5 approach may lack power to identify recombination between such closely related sequences (i.e., in the presence of little diversity), and while precautions were taken when running and analyzing results to reduce the risk of reporting false positives, caution should be applied when interpreting the donor/recipient relationship results for each supported recombination event (Table S5).

### ECoV site-specific natural selection inference

**Fig 5:**
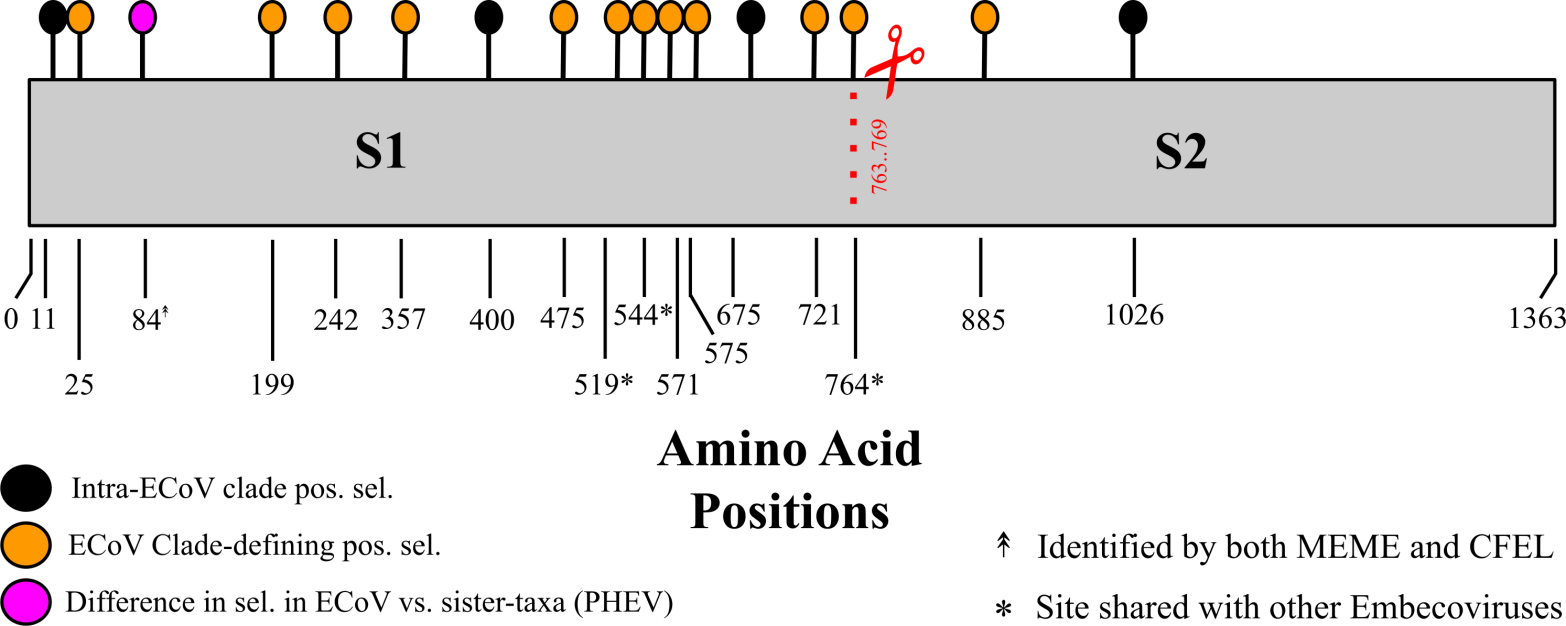
Sites under positive selection in the ECoV spike protein. Sites highlighted in black correspond to positively selected sites in an ECoV-specific analysis identified by the MEME method; orange sites indicate those under positive selection on the ancestral branch of ECoVs identified by MEME, and the magenta site indicates the site evolving differently between ECoVs and its sister-taxa (PHEV), identified by the Contrast-FEL method. Site 84 was identified by both the MEME and Contrast-FEL methods. Site 519 was also identified on the HKU23 (camel coronaviruses) ancestral branch, 544 on the HCoV-OC43 ancestral branch, and 764 (within the S1/S2 proteolytic cleavage site) on the HKU14 (rabbit coronaviruses) ancestral branch. Spike S1 and S2 subunits are separated by the putative proteolytic cleavage domain highlighted by both a red dashed line and scissors.

There was no evidence for genetic recombination within any of the following ECoV viral protein alignments analyzed: HE, S, E, M, and N. The MEME method (50) was applied to all branches in each RFP and associated phylogeny. We identified positive selection acting on codons in three of the five ECoV gene alignments analyzed: HE, S, and N (Table 3). Briefly, there were two sites detected in both HE and N, and five in Spike [four in subunit 1 (S1) and one site in S2, highlighted in black in Fig. 5].

**TABLE 3:** ECoV-specific positively selected sites^*a*^

Gene	Amino acid pos. in NC99 protein	Amino acid base composition	Protein subdomain	*P* value
HE	114	A_13_S_1_	n/a	0.018
HE	396	V_13_L_1_	n/a	0.042
Spike	11	T_14_G_2_	Signal peptide	0.002
Spike	84	E_11_A_2_G_2_Y_1_	A	0.008
Spike	400	S_13_N_3_	B	0.026
Spike	675	T_15_N_1_	CD	0.032
Spike	1026	G_11_V_5_	S2	0.032
N	135	A_18_V_3_	n/a	0.026
N	225	S_20_A_1_	n/a	0.034

^*a*^ Amino acid composition at the position in the alignment is reported with the single letter amino acid code and the count subscripted. *P* values are based on 500 parametric bootstrap replicates done separately for each site. Sites with *P* value ≤ 0.05 are reported. “*n/a*” in Protein subdomain column indicates that no specific protein subdomain is associated with the amino acid position identified.

### ECoV differentiating and *Embecovirus* clade-defining adaptive evolution

We generated an alignment using full-length Spike protein sequences from representative embecoviruses (see Materials and Methods for details). The presence of genetic recombination was inferred using the GARD method (47), which identified three supported breakpoints, resulting in four putatively RFPs (nucleotide breakpoint indices appear in Table S6). A maximum likelihood phylogeny inferred for each RFP was reconstructed with RAxML-ng (43), and subsequent natural selection analyses were performed on each RFP alignment and their respective phylogeny. PHEV is the sister-taxa to ECoV, so we used the Contrast-FEL method (51) to identify S codon positions evolving differently between ECoV and PHEV (see Fig. 2). Codon position 84 was the only site identified to be evolving differently (highlighted in magenta in Fig. 5). We used the MEME method (50) to identify positively selected sites on the ancestral branch of each *Embecovirus* clade represented in this study (see Materials and Methods for all 11 members represented). On the ancestral ECoV branch, a total of 12 sites were identified to be under positive selection, 11 of which fall in S1 and one that falls in S2 (highlighted in orange in Fig. 5). Three of the 12 sites were also identified to be selected on other ancestral member branches: 519, 544, and 764, shared with HKU23 (camel), HCoV-OC43 (human), and HKU14 (rabbit), respectively.

**Fig 6:**
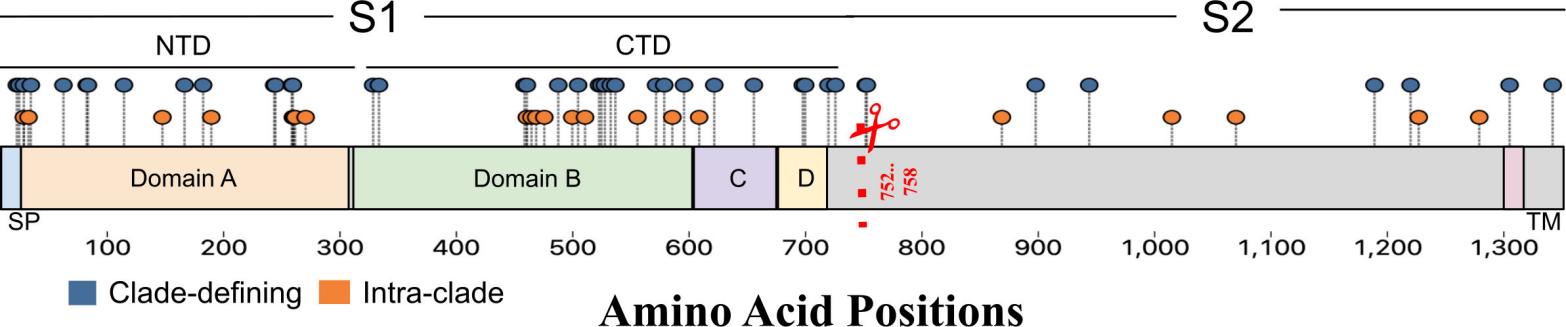
Clade-defining (blue) and intra-clade (orange) sites under positive selection identified in 825 HCoV-OC43 sequences, mapped to HCoV-OC43 (accession no. AAT84354.1). All sites mapped correspond to ungapped amino acid positions. The Spike protein is separated into S1 and S2, where S1 is subdivided into the NTD (Domain A) and CTD (Domain B, **C and D**). The signal peptide (SP), S1/S2 cleavage site (red dashed line and scissors), and transmembrane domain (TM) are all highlighted.

The largest number of selected sites inferred on an ancestral branch was inferred on that of HCoV-OC43 (42 total). Of those 42 sites, 16 fell within the N-terminal domain (NTD) of S1, 18 in the C-terminal domain (CTD) of S1, and 8 sites in S2 (Fig. 6). We also inferred intra-specific positive selection in HCoV-OC43, that is, the sites subject to selection on branches within the HCoV-OC43 clade (highlighted in orange in Fig. 6 and in Table S7). The fewest number of inferred sites under selection were observed on the HCoV-HKU1 ancestral branch, with only two sites identified. The remaining number of inferred sites subject to positive selection on *Embecovirus* member ancestral branches were as follows: CRCoV: 21; HKU14 and MHV: 19; BCoV: 7; PHEV: 12; HKU23 and HKU24: 11; and longquan R1 rat coronavirus: 8 (Table S8).

## DISCUSSION

Horses, a common husbandry and companion animal, carry important zoonotic diseases such as West Nile Virus (58), Hendra virus (59), and ECoV, all of which can have significant negative effects on infected animals and humans. When animals come into close contact with people, whether as companion animals or wildlife, they can act as reservoirs for viral recombination and spillover. As equids are transported around the world to participate in competitions, so are the pathogens they harbor (14, 60, 61), which could ultimately pose a threat to both naive animal and human hosts. Beyond ECoV, the horses analyzed herein harbored other viruses and parasites (Table S9). ECoV appears to have a global distribution, with viral isolates from distant geographic regions sometimes clustering together (Fig. 3). Relatively little is known about the evolutionary forces that shape ECoVs, a *Betacoronavirus* in subgenus *Embecovirus*, as there have been no systematic natural selection analyses performed on this clade to date. Since only one publicly available full-length ECoV genome sequence from a horse isolated from the United States of America (USA) existed prior to our study, there is limited knowledge of how ECoV sequences in the USA are evolving and spreading. Natural selection analyses of geographically localized coronaviruses have shed light on unique, region-specific patterns of evolution (62, 63). Within embecoviruses, there is substantial evidence that tropism shifts (between tissue type as well as between species) occurred often, suggesting that the ancestral *Embecovirus* may have been a highly adaptable generalist virus. For example, HCoV OC43 (HCoV-OC43) has a complex recombinant history involving origins in bovines, camels, rabbits, canines, equines, and mice (2). Here, we contribute four ECoV genome assemblies (along with their respective RNAseq data sets) isolated from horses from the USA and perform comprehensive evolutionary testing to identify ECoV-specific signatures of evolution. Further, we examine *Embecovirus* clades to infer patterns of evolution associated with each individual clade and highlight significant differences in the evolutionary history of the two human host embecoviruses, HCoV-OC43 and HCoV-HKU1.

### ECoV-specific signals of genetic recombination and quasispecies development

Since a great deal of CoV genetic diversity derives from the process of recombination, which in some cases has given rise to CoV spillover events (24, 64–69), we tested for evidence of genome-wide ECoV-specific recombination using 14 available ECoV genomes and identified 12 supported genetic recombination events using RDP5 (39) (see Materials and Methods for details) (Fig. 4). All but one of these events fell within ORF1ab, with the remaining event starting in S2 of Spike and continuing until the end of the viral genome. Of note, the overwhelming majority of genetic recombination events observed within the ECoV genomes analyzed deviate from previously described genome-wide coronavirus recombination patterns, as these events fall within “cold-spots” of the genome (genomic regions where recombination events tend not to occur) (23). Further, due to CoV genetic diversity, what evolutionary patterns hold for one clade need not hold for another. Analyses of related CoVs suggest that the ORF1ab polyprotein may be involved in viral replication and pathogenesis (70). Given that genetic recombination events within and between CoVs have resulted in novel virus phenotypes, it is possible that the genetic recombination patterns observed in ECoVs could have a similar impact.

Quasispecies development is another mechanism for creating genetic diversity in ECoVs. As the virus infects a host, intra-host evolution may create multiple viral populations, possibly with altered phenotypes. Intra-host coronaviral evolution in humans has been implicated in the development of virulent mutations (21, 22, 71–73), and in animals has yielded viruses with altered tissue specificity (74–76). Quasispecies development in CoV, another member of the *Embecovirus* group, has been shown to be a major contributing factor for the ability of the virus to colonize different host tropisms (77). Although we followed a conservative, published protocol with strict thresholds for detecting intra-host variants (mAF between 10% and 90%, and more than 10 reads supporting the variant) (33), the intra-host variant results reported herein should be interpreted with caution. Two of the four horse data sets each contained single nucleotide polymorphic sites that passed the threshold metrics, and both fell within the nucleocapsid (N) gene (Table 2.). Horse 1 was the oldest horse out of the four (over 27 years old), and the nucleotide mutation identified in this sample fell within codon 15 and resulted in a non-synonymous (S15F) mutation. Horse 4 was the second oldest horse and presented with respiratory clinical symptoms, where the nucleotide mutation identified fell within codon 185 of the N gene and resulted in a synonymous (Y185Y) mutation. The intra-host variant from Horse 1 along with the codon subject to positive selection in the intra-ECoV selection analysis (amino acid pos. 135) fell within the N-NTD, and the intra-host variant from Horse 4 along with intra-ECoV positively selection codon position 225 fell within a central linker region. For HCoV-OC43, it has been shown that the N-terminal domain of N (NTD-N) (aa 1–173) acts as an RNA-binding scaffold (78), which may play a role in trafficking the virus to the host nucleus (79). The N protein also functions to package the viral genome; critical to the success of viral self-assembly (80). Reliably identifying *bona fide* quasispecies remains an open problem. Because there are several different approaches to detect viral quasispecies (81), there tends to be a wide range of thresholds and conditions reported as evidence for quasispecies. For example, minority viral populations have been reported at as low as 1% allele frequency (21), yet it has been demonstrated that mutations detected at low frequencies (<2%) in RNA viruses may be artifacts of the sample preparation or sequencing process (82). Here, we took a more conservative approach and reported any mAF ≥10% to focus on positions more likely to be associated with advantageous mutations (83); due to computational reproducibility of our analysis the exact mAF threshold can be adjusted to report variants at other mAFs. Nonetheless, it is highly desirable to collect and analyze more intra-host ECoV sequence data before a stronger association between intra-host evolution and ECoV infection can be made. However, since our samples had very high viral loads, and because our method does not require DNA amplification, many of the common sources of error are not applicable.

### ECoV-specific signatures of natural selection

Signals of adaptive evolution were inferred in the ECoV Spike protein, predominantly within S1. The S1 subunit can be further subdivided into functional protein subdomains, starting with the signal peptide (SP). The SP is the first 15 amino acids at the N-terminus of S1 in the related *Embecovirus*, HCoV-OC43 (84), and functions to aid in various secretory viral protein pathways (85, 86). We identified signals of selection in the putative ECoV SP at site 11, falling within the H-region of the SP structure (86). This alpha helix region forms a hydrophobic core, and the hydrophobicity of this region can impact SP conformation, SP cleavage from the mature protein, secretion pathway used, and protein processing (86)—all of which can alter viral efficiency. We identified an adaptively evolving site within the SP of the Spike protein from a recent coronavirus zoonotic spillover event involving canines and humans (CCoV-HuPn-2018) (29). Positive selection in signal peptides is not widely reported, most likely because it is rarely examined. We propose that adaptive evolution in coronavirus Spike SP could be an underappreciated aspect of Spike functional refinement and host adaptation.

The next downstream proximal subdomain to SP in embecoviruses is Domain A, also contained within the NTD of S1. The NTD can act as a receptor-binding domain, binding proteins or sugars (87). Indeed, sugar binding has been identified in related embecoviruses, BCoV (88), HCoV-OC43 (84), and a newly emerged Camel coronavirus (HKU23) (89). Within HKU23, several positions were identified as critical to sugar binding, including positions 81 and 83, forming a sugar-binding pocket (90). There was a singular site of differential selection pressure of ECoVs relative to all other embecovirses analyzed herein, site 84. Given the homology between HKU23 and ECoV at this location in Spike, it is reasonable to hypothesize that this site in ECoVs may play a role in viral receptor specificity and/or binding with regard to sugars. A recent study examining antibody binding in HCoV-OC43 observed that a group l antibody (46C12) bound to the NTD (site 81) of HCoV-OC43, and showed that the antibody used molecular mimicry to bind to the sugar-binding motif within the NTD (91). Diversifying selection at site 84 in ECoVs may serve several purposes; to bind sugars and/or evade the host immune system.

Downstream of domain A, are the remaining domains B, C, and D, collectively referred to as the C-terminal domain (CTD) of S1. In embecoviruses the CTD of S1 contains the protein receptor binding motif (RBM) (92) and is housed within domain B. In HCoV-HKU1, regions of the CTD have been identified as responsible for protein receptor binding and epitope neutralization (93). The specific RBM within HCoV-HKU1 was identified within the spike insertion loop, consisting of five beta sheets and five helices (429–588) (93). Within that homologous stretch in ECoV, there are six sites subject to selection on the ECoV ancestral branch: 457, 519, 538, 571, 575, and 721 (Table S8). While the specific host receptor(s) that ECoVs interact with are not known, it could be that the selection observed within the CTD impacts receptor binding and antibody escape.

### Adaptive evolution differences in Spike of embecoviruses that have colonized the human host

We inferred clade-defining signatures of site-specific positive selection in the Spike protein for all *Embecovirus* clades analyzed herein (Table S8) (see Materials and Methods for details). Animal origins entangle the evolutionary history of all coronaviruses known to infect humans (2, 94). HCoV-OC43 and HCoV-HKU1 are no exception, as HCoV-OC43 shares a complex evolutionary history that may have involved bovines, rabbits, camels, dogs, pigs, and horses, and HCoV-HKU1 with mice (2). After the jump from animals, differences in within-viral species signals of adaptation have been reported in HCoV-OC43 and HCoV-HKU1 in response to human host colonization (95). Here, we individually inferred selection on the ancestral branch leading to HCoV-OC43 and to HCoV-HKU1 to identify the selection pressure associated with the shift from ancestor to the human host, in effect excluding evolutionary signals that may come from circulation within the human host. The highest (42) and lowest (2) number of sites subject to positive selection on *Embecovirus* member ancestral branches were observed for HCoV-OC43 and HCoV-HKU1, respectively. The sites identified in HCoV-OC43 are scattered across the NTD and CTD of Spike (highlighted in blue in Fig. 6) in regions associated with sugar binding, immune escape, and protein receptor binding (84, 91, 93). The two sites identified in HCoV-HKU1 fall within S2 (Table S6). Of note, HCoV-OC43 appears to have a far more complicated evolutionary history, that implicates multiple different ancestral animal hosts prior to its jump into humans relative to HCoV-HKU1. Specific functional differences between the HCoV-OC43 and HKU1 Spike proteins may also play a role in the differences in evolutionary adaptation from ancestral host to humans. For example, a recent study reported on the nuanced differences in conformational changes that both HCoV-HKU1 and HCoV-OC43 undergo throughout the receptor-binding process during host cellular entry (96). This suggests that CoVs that circulate in certain animals may have subtly different functional roles in each host, which may result in an altered predisposition of spillover into the human host.

In conclusion, we contribute four novel ECoV genome assemblies (and their respective RNAseq data sets), as well as report on the evolutionary history of ECoVs and other embecoviruses, in particular those that have colonized the human host. We highlight the potential significance of genetic recombination in ORF1ab (9), as well as the potential presence of viral quasispecies in ECoV samples. We also highlight the difference in natural selection on the ancestral branch of the two HCoV embecoviruses sampled to date; with large numbers of positively selected sites in Spike in HCoV-OC43 (42) and only one in HCoV-HKU1, potentially reflective of their ancestral animal host and spike protein functional differences.

## Data Availability

The four novel genome assembly accession numbers (Horse 1: OR468097, Horse 2: OR468098, Horse 3: OR468099, Horse 4: OR468100) and respective RNAseq data sets (Horse 1: SAMN36993381, Horse 2: SAMN36993382, Horse 3: SAMN36993383, Horse 4: SAMN36993384) are publicly available on GenBank under BioProject: PRJNA1005627.

## SUPPLEMENTAL MATERIAL

Fig. S1 spectrum.00867-24-s0001.pdf SimPlot++ NS2 ECoV comparison.

Table S1 spectrum.00867-24-s0002.csv Accession numbers for ECoV proteins used.

Table S2 spectrum.00867-24-s0003.csv Embecovirus accession numbers.

Table S3 spectrum.00867-24-s0004.csv Number of unique viral sequences kept for analyses.

Table S4 spectrum.00867-24-s0005.csv Pairwise distance matrix estimated using MEGA11 with amino acid NS2 sequences.

Table S5 spectrum.00867-24-s0006.csv RDP5 recombination results for ECoV genomes.

Table S6 spectrum.00867-24-s0007.csv GARD nucleotide breakpoints inferred from the Embecovirus spike analyses.

Table S7 spectrum.00867-24-s0008.csv Intra-selection analyses for HCoV-OC43 and HKU1.

Table S8 spectrum.00867-24-s0009.csv Positive selection at sites inferred on clade-defining branches.

Table S9 spectrum.00867-24-s0010.csv Non-ECoV taxa identified in equines from this study.

ASM does not own the copyrights to Supplemental Material that may be linked to, or accessed through, an article. The authors have granted ASM a non-exclusive, world-wide license to publish the Supplemental Material files. Please contact the corresponding author directly for reuse.
