# Supplementary material for: Positive selection, genetic recombination, and intra-host evolution in novel equine coronavirus genomes and other members of the Embecovirus subgenus
Source: Microbiol Spectr. 2024 Oct 7;12(11):e00867-24. doi: 10.1128/spectrum.00867-24 (PMC11542594; doi:10.1128/spectrum.00867-24)
Supplement: Fig. S1 — SimPlot++ NS2 ECoV comparison. [file spectrum.00867-24-s0001.pdf]

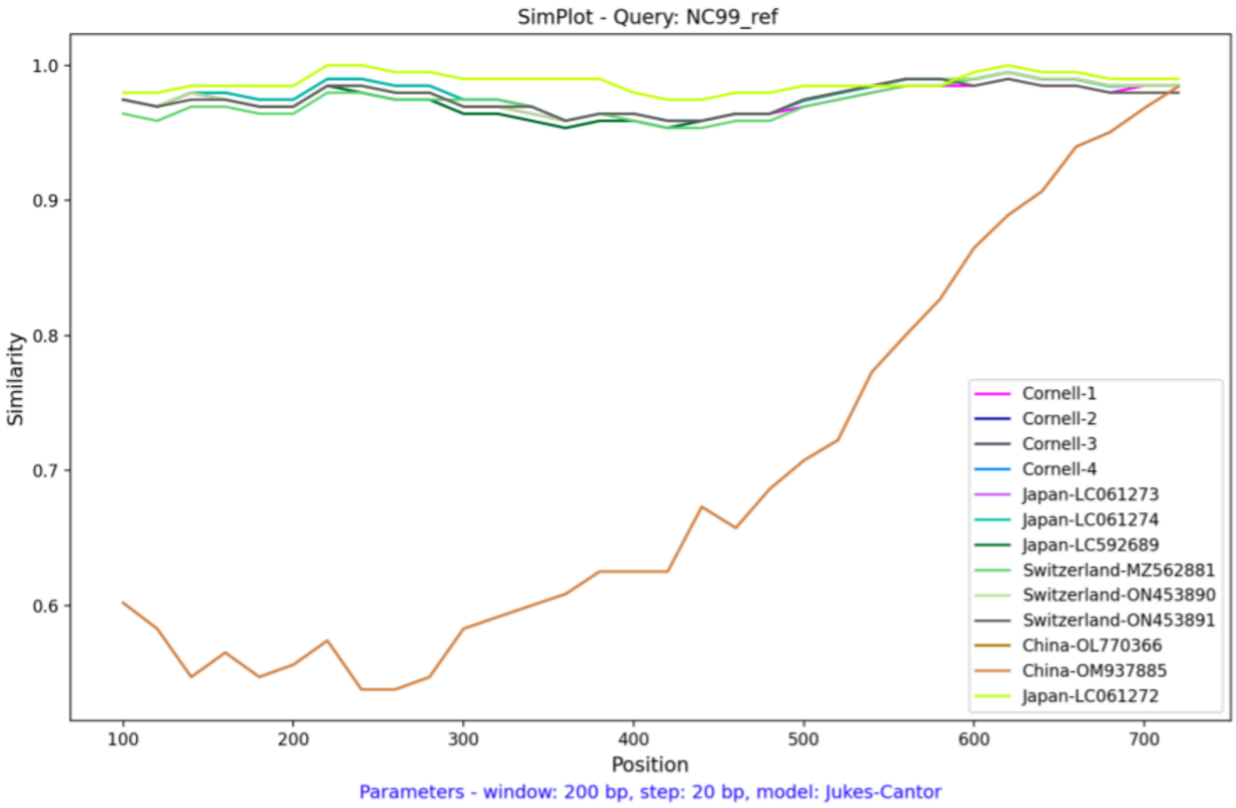

FIG S1 Sequence similarity as identified by SimPlot++ for the NS2 protein sequences from the 14 ECoV genomes analyzed herein. NC99 was used as a reference sequence. The two sequences isolated from China were identical, thus appearing as a singular line (brown). All other NS2 sequences analyzed appear to be similar to one another.
